# Effect of extracorporeal shock wave therapy on nerve conduction: a systematic review and meta-analysis

**DOI:** 10.3389/fneur.2024.1493692

**Published:** 2024-11-22

**Authors:** Liuxin Yang, Xuan Li, Shuhan Li, Jiao Yang, Dianhuai Meng

**Affiliations:** Rehabilitation Center, The First Affiliated Hospital with Nanjing Medical University, Nanjing, Jiangsu, China

**Keywords:** physical therapy modalities, electrodiagnosis, nerve conduction studies, peripheral nerve injuries, review

## Abstract

**Background:**

Extracorporeal shock wave therapy (ESWT), as a non-invasive physical agent modality, was effective in relieving spasticity, reducing pain, and improving dysfunction. This systematic review and meta-analysis aimed to investigate the effect of ESWT on nerve conduction, and to find out whether the ESWT group is superior to the control or other comparison groups, thus providing support for guiding the rehabilitation of peripheral nerve injury in clinical work.

**Methods:**

PubMed, Web of Science, the Cochrane Library, and Embase were searched from inception to August 20, 2024. This review adhered to the Preferred Reporting Items for Systematic Reviews and Meta-Analyses (PRISMA) guidelines and registered in the PROSPERO database (registration number CRD42024500891). It aimed to compare (1) the ESWT group (ESWT) and baseline, and (2) subgroup analyses: ESWT and the control group (Control), ESWT and the local corticosteroid injection group (LCI), ESWT combined with physical therapy (ESWT + PT) and PT alone, and ESWT and PT. Outcome indicators extracted were nerve conduction study results: sensory nerve action potential (SNAP) amplitude, SNAP distal latency, sensory nerve conduction velocity (SNCV), compound muscle action potential (CMAP) amplitude, motor nerve distal latency (MNDL), motor nerve conduction velocity (MNCV), H/M ratio and H-reflex latency.

**Results:**

A total of 879 papers were identified through the database searches. Twenty-four trials were included in the systematic review, and 22 trials were included in the meta-analysis. The results showed that: (1) compared to the baseline, ESWT reduced SNAP distal latency mid-term (MD, −0.39; 95% CI: −0.52, −0.26; *I*^2^ = 85%), and improved SNCV both short-term (MD, 4.36; 95% CI: 1.23, 7.49; *I*^2^ = 91%) and mid-term (MD, 2.65; 95% CI: 1.79, 3.51; *I*^2^ = 61%). At the same time, it reduced MNDL short-term (MD, −0.61; 95% CI: −0.91, −0.30; *I*^2^ = 92%), but had no significant effect on MNCV. (2) Subgroup analyses showed that ESWT was superior to Control but not significantly better than LCI, especially in SNCV. The excitatory effect of ESWT + PT on the sensory and motor nerves was significantly better than that of PT alone.

**Conclusion:**

ESWT had some excitatory effect on peripheral nerves, especially on sensory nerve studies. Although the efficacy of this treatment was superior to that of the control group, and the combined treatment with PT was more effective than PT alone, its efficacy might not exceed that of LCI.

**Systematic review registration:**

Unique identifier: PROSPERO (CRD42024500891).

## 1 Introduction

Shock wave (SW) is a kind of acoustic pulse with unique physical characteristics, which is characterized by high peak pressure, fast rise rate, short duration, and low stretch amplitude (1). There are three different shockwave generator technologies used today: electrohydraulic (EH), electromagnetic (EM), and piezoelectric (PE). ESWT promotes biological and neurological effects through a combination of mechanical conduction, angiogenesis, vacuolation and biochemical signals. Common extracorporeal shock wave therapy (ESWT) in clinical practice is divided into focused ESWT (fESWT) and radial ESWT (rESWT). As a non-invasive physical agent modality, ESWT has the advantages of safety, effectiveness, and ease of operation. Currently, it has been widely used for urolithiasis (2), musculoskeletal diseases (3) and chronic pain (4). Additionally, skin ulcers, detrusor underactivity, erectile dysfunction, and chronic prostatitis (CP)/chronic pelvic pain syndrome (CPPS) (5) have gradually become research hotspots. Notably, the latest guidelines from the International Society for Medical Shockwave Treatment (ISMST) (6) state that spasticity, polyneuropathy, and carpal tunnel syndrome (CTS) are expert indications. However, in terms of the nervous system, peripheral nerve lesions and pathologies of the spinal cord and brain are still experimental indications. Extracorporeal shock wave in nerve applications requires further research.

As a form of electrodiagnosis, nerve conduction studies play an important role in the diagnosis and evaluation of nerve and muscle lesions (7). The functional status of the peripheral nerves is evaluated and analyzed mainly according to the principles of neuroanatomy and neuroelectrophysiological characteristics, providing a basis for further clinical diagnosis (8). The measured values included motor conduction studies and sensory conduction studies, both of which included amplitude, latency, and conduction velocity, as well as late responses (F response and H reflex), blink reflex, and repetitive nerve stimulation.

Previous reviews had shown that ESWT was effective in relieving poststroke spasticity (9), reducing pain (4, 10), and improving dysfunction (11), mainly according to the patient scores on clinical scales, such as the Visual Analogue Scale (VAS) and the Boston Carpal Tunnel Questionnaire (BCTQ). Although there had been some related studies on the effect of ESWT on nerve conduction studies (12–14), most of them only selected a few indicators (amplitude, latency, or conduction velocity) for analysis. Additionally, the results of these studies were contradictory, which brought inconvenience and confusion to their clinical applications.

This review had significant advantages over previous systematic reviews. Participants with any disease, except contraindications, were eligible for inclusion in this study, thus expanding the scope of the research. Additionally, broader indicators of nerve conduction were included. We aimed to conduct a comprehensive investigation into the potential excitatory or inhibitory effects of ESWT on nerve conduction and to determine whether ESWT is superior to Control (including no intervention, sham ESWT, or conservative exercise) or other comparison groups, such as LCI or PT.

## 2 Methods

This systematic review adhered to the Preferred Reporting Items for Systematic Reviews and Meta-Analyses (PRISMA) guidelines and recommendations of the Cochrane Handbook for Systematic Reviews of Interventions. The protocol was registered in the PROSPERO database (registration number CRD42024500891).

### 2.1 Search strategy

Data were searched up to August 20, 2024, using four databases: PubMed, Web of Science, the Cochrane Library, and Embase. The keywords and search strategy include: (“extracorporeal shockwave” OR “extracorporeal shock wave” OR “ESWT” OR “ESW”) AND (“nerve” OR “nerves”). The reference lists of the relevant articles and reviews were evaluated to identify the potentially eligible studies. LY conducted the topic search, and XL and JY further screened against the criteria.

### 2.2 Study selection

The studies included in the meta-analysis were required to meet the following PICOS criteria: (1) participants: included participants with various types of neurological injuries of any age, sex, and disease stage; (2) intervention: rESWT or fESWT; (3) comparator: compared (3.1) the ESWT group (ESWT) and baseline, and (3.2) subgroup analyses: ESWT and the control group (Control), ESWT and the local corticosteroid injection group (LCI), ESWT combined with physical therapy (ESWT+PT) and PT alone, and ESWT and PT; (4) outcomes analyzed: nerve conduction studies results, including measurements of sensory nerve action potential (SNAP) amplitude, SNAP distal latency, sensory nerve conduction velocity (SNCV), compound muscle action potential (CMAP) amplitude, motor nerve distal latency (MNDL), motor nerve conduction velocity (MNCV), H/M ratio and H-reflex latency; and (5) study design: randomized controlled trials. Exclusion criteria: (1) recent treatment involving physical agents and neurotrophic drugs; (2) presence of metal or electronic devices in the body, such as pacemakers, neurostimulators, and medical pumps; (3) combined with infectious, rheumatological, and immunological diseases, such as respiratory, urinary, skin and soft tissue infections, systemic lupus erythematosus and desiccative syndrome; (4) combined with bleeding diseases and thrombosis in the treatment area; (5) history of soft tissue injury, extensive scarring and skin grafts in the detected or intervened limb; (6) type of literature review, meta-analysis, case report, guideline, letter, book, note and conference abstract. Literature searches, screening, and selection of studies were performed independently by two reviewers. In the event of disagreements, a third researcher made the final decision.

### 2.3 Outcomes

The main measurements were nerve conduction studies results, including SNAP amplitude, SNAP distal latency, SNCV, compound muscle action potential (CMAP) amplitude, MNDL, MNCV, H/M ratio, and H-reflex latency. Based on a previous systematic review (12), we defined short-term as within 1 month after therapy (≤1 m), medium-term as 1–6 months after therapy, and long-term as 6 months or more (≥6 m) after therapy.

### 2.4 Data extraction

After removing duplicates, two authors independently assessed the studies that met the inclusion criteria. If a related outcome was either unavailable or incomplete in a published article, we attempted to contact the corresponding author for the original data. The following information was extracted: author, publication year, study design, participants (number, age, sex, types of disease), interventions (fESWT/rESWT, frequency, the number of extracorporeal shock wave pulses, intensity, energy flux density, and treatment sessions), outcome measures (nerve conduction studies results and other clinical evaluation indicators), follow-up, and side effects. In the analysis of data from nerve conduction studies, we first unified the different representations in the literature by grouping different terms that express the same test results into a single category. For example, the “SNAP distal latency” and “SNDL” are both considered to be the same type of data. Next, we systematically collected and collated the data of all experimental and control groups at baseline and during follow-up, and changes in data after the follow-up period, which were expressed as the mean and standard deviation (SD). Only a few studies had been reported as median (15) or standard error (16, 17). We converted these values to mean and SD using the methods described in the Cochrane Handbook (18).

### 2.5 Risk-of-bias assessment

The risk of bias in the included studies was assessed by two researchers (LY and XL) according to the RoB 2.0 (19). Any disagreements between assessors should be resolved through discussion by a third researcher (SL). This risk assessment included randomization process, deviations from intended interventions, missing outcome data, measurement of the outcome, and selection of the reported result. There were several different signaling questions under each domain. Researchers need to make judgments and answer these questions objectively when evaluating the risk of bias in RCTs. The signaling questions generally had five alternative answers: yes (Yes, Y), probably yes (Probably Yes, PY), probably no (Probably No, PN), no (No, N), and no information (No Information, NI). Studies were categorized as low risk, some concerns, or high risk.

### 2.6 Certainty of evidence

The Grading of Recommendations Assessment, Development and Evaluation (GRADE) tool was used to judge the certainty of evidence. Based on the risk of bias, inconsistency, indirect evidence, imprecision, and publication bias, we graded the certainty of the evidence on four scales: high, moderate, low, or very low.

### 2.7 Data analysis

Statistical analyses were performed using the RevMan 5.4 software. All study outcomes analyzed in this article were continuous data. The summary results of all trials were reported using the mean difference and 95% confidence intervals (CIs). *Q* test and *I*^2^ test were used to analyze heterogeneity among the studies (20). If *p* < 0.1 or *I*^2^ > 50%, indicating high heterogeneity, the random-effects model was used for analysis. In contrast, if *p* ≥ 0.1 or *I*^2^ ≤ 50%, indicating low heterogeneity, a fixed-effects model was used. As the control interventions varied among trials, we performed a subgroup meta-analysis to compare them: (1) ESWT vs. Control, (2) ESWT vs. LCI, (3) ESWT + PT vs. PT, and (4) ESWT vs. PT. Descriptive analyses were performed for the data that were not included in the meta-analysis. Statistical significance was set at *p* < 0.05.

Sensitivity analyses and publication bias assessments were performed on the outcomes of the electrodiagnostic studies before and after ESWT. Considering the small sample size and the limited number of studies included in the subgroup analyses, we did not conduct both of them on the results of the subgroup analyses. The leave-one-out sensitivity analysis was used to evaluate the stability of the meta-analysis results, and a funnel plot was used to assess the risk of publication bias. If the funnel plot was symmetrical, no significant publication bias would be observed. Some modifications were made to the protocol. To improve the quality of evidence and reduce the risk of bias, only RCTs were included in this review, and no observational studies were included. The subgroup analyses did not follow the predefined settings in the review protocol but were based on the information provided by the included trials and data analysis.

## 3 Results

### 3.1 Study selection

**Figure 1:**
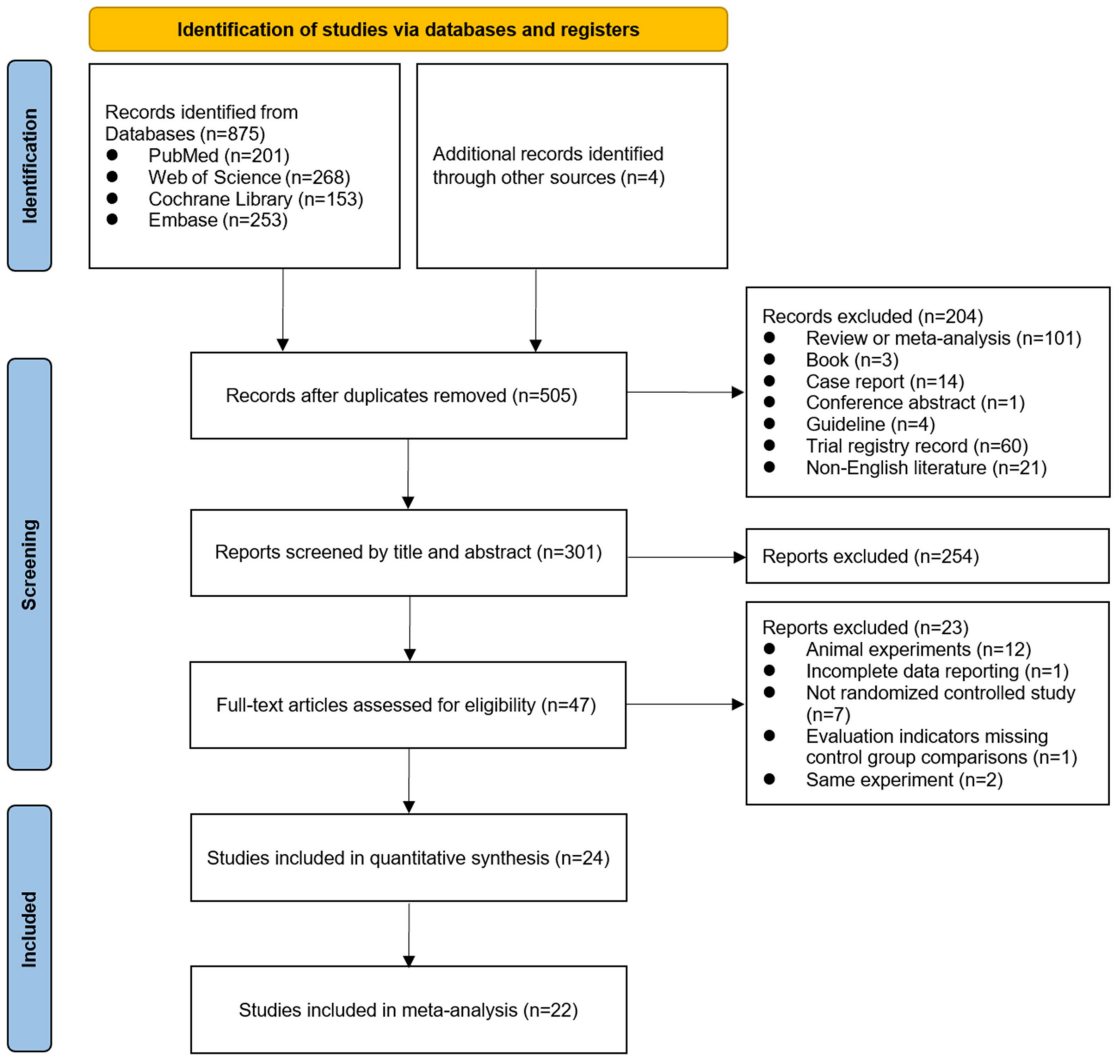
Preferred Reporting Items for Systematic Reviews and Meta-Analysis (PRISMA) flow diagram of study selection process.

A total of 879 papers were included from searching four major literature databases and reviewing the literature included in meta-analyses related to ESWT in recent years. Search strategies for each database and registry could be found in Supplementary material S1. Twenty-four articles (13, 15–17, 21–40) were included in the systemic review after excluding duplicates, limiting the types of literature, screening titles and abstracts, and assessing the eligibility of full texts (Figure 1) (the specific reasons for excluding trials were shown in Supplementary material S2). Based on treatment-specific comparative grouping, these 24 studies were divided into four groups: ESWT vs. Control (13, 15, 16, 24, 26, 27, 31, 32, 36, 37, 40) (*n* = 11), ESWT vs. LCI (13, 22, 23, 34, 35, 38) (*n* = 6), ESWT + PT vs. PT (15, 21, 25, 28, 33, 39) (*n* = 6), and ESWT vs. PT (30, 32) (*n* = 2). In addition, there is one study (29) comparing ESWT with nutraceutical and the other (17) comparing the combined treatment of platelet-rich plasma and rESWT with platelet-rich plasma alone. Among them, The Control group included no intervention, sham ESWT, and conservative exercise. PT included therapeutic exercise, conservative treatment, sensory re-education program, nerve mobilization, ultrasound, vibrator and transcutaneous electrical nerve stimulation (TENS). Splint was considered a routine treatment and not a comparator.

### 3.2 Characteristics of included trials

Characteristics of included trials are provided in Table 1. A total of 1,445 subjects were enrolled, of which 297 (17.2%) were male, 921 (65.9%) were female, and 227 (16.9%) did not report their gender. These trials were published between 2015 and 2024. The average age of the participants was 50.38 years old, with two papers (13, 28) not counting the age of the subjects. Out of the 24 RCTs analyzed, 19 focused on CTS, one on children with cerebral palsy, one on post-burn TTS, one on poststroke plantar flexor spasticity, one on poststroke spastic equinus, and one on diabetic neuropathic foot. In instances where multiple time measurement points exist within a specified temporal segment (short-term, medium-term, or long-term), the standard practice is to select the time point with the longest duration within that range (9). Similar treatment groups in the same study (e.g., ESWT treatment groups with different treatment parameters) were combined into one treatment group for analysis. The evaluation outcomes were divided into two categories: subjective and objective. Subjective evaluation outcomes include questionnaires or scales that reflect patients’ feelings and patient-perceived global function, such as VAS, BCTQs, BCTQf, hand grip strength, and so on. This review, however, mainly focused on the objective evaluation indicators related to nerve conduction studies, covering a total of 8 indicators. Among them, SNAP amplitude was evaluated in 11 articles, SNAP distal latency in 11 articles, compound muscle action potential (CMAP) amplitude in 12 articles, MNDL in 17 articles, SNCV in 14 articles, MNCV in 4 articles, H/M ratio in 3 articles and H-reflex latency in 1 article. In assessing the SNAP amplitude and SNCV, Zhang et al. (39) measured both thumb-wrist and middle finger-wrist. We divided them into groups a and b, and included them in the statistical analyses separately.

**Table 1:** Characteristics of the included trials.

Study	Sample size		Age		Gender (M/F)		Population	Nerve	Interventions	Outcomes		Measure time (electrodiagnostic studies)	Side effect
	ESWT	Control	ESWT	Control	ESWT	Control				Electrodiagnostic studies	OOther		
Abdel et al. 2015	15	15	5.75 ± 0.51	5.83 ± 0.34	6/9	6/9	Cerebral palsy children	Tibial nerve	Experimental group: ESWT + therapeutic exercises 2000 shots, 0.32 mJ/mm^2^, 3 sessions/week for 1 week (700/session) Control group: therapeutic exercises	H/M ratio	Gait measurements (speed, cadence, stride length, single limb support, double limb support and ankle dorsiflexion in gait cycle)	Baseline, 4 w after treatment	NR
Ahmed et al. 2021	20	20	51 ± 6	49 ± 8	4/16	5/15	CTS	Median nerve	Experimental group: rESWT 5,000 shots, 15 Hz, 4 bar, once/week for 3 weeks Control group: LCI 1 mL of triamcinolone 10 mg mixed with 1 mL of 1% lidocaine, 1 session	Peak sensory distal latency, SNAP amplitude, SNCV, CMAP amplitude, MNDL, MNCV	VAS, BCTQ	Baseline, 3 m after treatment	No
Atthakomol et al. 2018	13	12	46 ± 9	53 ± 12	5/8	1/11	CTS	Median nerve	Experimental group: rESWT 5,000 shots, 15 Hz, 4 bar,1 session Control group: LCI 1 mL of triamcinolone 10 mg mixed with 1 mL of 1% lidocaine, 1 session	SNAP amplitude, peak sensory distal latency, CMAP amplitude, MNDL	BCTQ, VAS	Baseline, 12 w after treatment	The rESWT group: a few patients mentioned minimal pain during treatment
Chang et al. 2020	32	32	56.47 ± 1.41	58.63 ± 1.72	3/29	2/30	CTS	Median nerve	Experimental group: 1 dose of ultrasound-guided PRP injection + rESWT 2000 shots, 5 Hz, 4 bar, 1 session Control group: 1 dose of ultrasound-guided PRP injection + Sham rESWT	SNCV, MNDL	BCTQs, BCTQf, CSA	Baseline, 1 m, 3 m, and 6 m post-PRP injection	No
Gesslbauer et al. 2021	10	10	55.8 ± 4.66	54.0 ± 17.4	2/8	4/6	CTS	Median nerve	Experimental group: fESWT + splint 500 shots, 4 Hz, 0.05 mJ/mm^2^, once/week for 3 weeks Control group: Sham fESWT + splint	SNCV, MNDL	VAS, hand grip strength, BCTQs, BCTQf, SF-36 health survey	Baseline, 12 w after treatment	No
Habibzadeh et al. 2022	Point SW: 20 Sweep SW: 20	20	Point SW: 45.40 ± 11.49 Sweep SW: 50.55 ± 11.99	51 ± 7.77	Point SW: 2/18 Sweep SW: 2/18	5/15	CTS	Median nerve	Experimental group: rESWT + PT + splint Point SW: 1,500 shots on the carpal tunnel, 4 sessions in 3 weeks Sweep SW: 1500 shots on the carpal tunnel and median nerve pathways, 4 sessions in 3 weeks Control group: PT + splint	SNAP distal latency, MNDL	VAS, BCTQs, BCTQf	Baseline, 1 w and 4 w after treatment	The rESWT group: transient pain and redness of the skin
Karatas et al. 2019	29	20	51.28 ± 9.16	51.65 ± 7.37	2/27	2/18	CTS	median nerve	Experimental group: ESWT + splint 1,500 shots, 0.1 mJ/mm^2^, 3 sessions/week for 3 weeks Control group: Sham ESWT + splint	SNAP distal latency, SNCV, CMAP amplitude, MNDL	VAS, BCTQs, BCTQf, hand grip strength	Baseline, 3 m after treatment	No
Ke et al. 2016	Group A: 30 Group B: 29	Group C: 30	Group A: 56.33 ± 1.48 (37–71) Group B: 55.45 ± 1.38 (40–68)	Group C: 58.13 ± 1.13 (45–66)	Group A: 6/24 Group B: 6/23	Group C: 5/25	CTS	Median nerve	Experimental group: rESWT Group A: 2000 shots, 5 Hz, 4 bar, 3-session (once weekly) + splint Group B: 2,000 shots, 5 Hz, 4 bar, 1-session + splint Control group: Sham rESWT + splint Group C: 3 sessions of sham rESWT + splint	SNCV	BCTQs, BCTQf, CSA	Baseline, 4 w, 10 w, and 14 w after the first session	No
Menekseoglu et al. 2023	27	28	43.8 (8.3)	46.9 (9.3)	NR	NR	CTS	Median nerve	Experimental group: rESWT + splint + exercise 2,000 shots, 1.6 bar, 6 Hz, once/week for 3 weeks Control group: Sham rESWT + splint + exercise	SNAP amplitude, SNAP distal latency, SNCV, CMAP amplitude, MNDL, MNCV	VAS, BCTQ, LANSS	Baseline, 1 m after treatment	The rESWT group: 3 participants experienced paresthesia symptoms along the median nerve trace
Mowafy et al. 2020	20	20	NR	NR	NR	NR	Post-burn TTS	Posterior tibial nerve	Experimental group: ESWT + PT 100 impulses/cm^2^, 6 Hz, 1.5 bar, every 2 weeks for 3 months Control group: PT	SNAP distal latency, MNDL	No	Before and after treatment	NR
Nada et al. 2023	50	50	55.35 ± 6.22	55.65 ± 6.0	35/15	30/20	Poststroke spastic equinus	Tibial nerve	Experimental group: rESWT 1,500 pulses, 0.10 mJ to 0.3 mJ/mm^2^, 4 Hz, once weekly for 1 month Control group: Sham rESWT once weekly for 1 month	H/M ratio	MAS, passive ankle dorsiflexion motion, 10 meters walk test	Baseline, immediately and 1 m after treatment	NR
Notarnicola et al. 2015	34	26	57.1 ± 9.5	60.2 ± 6.6	NR	NR	CTS	Median nerve	Experimental group: ESWT + splint + PT 1,600 shots, 4 Hz, 1.5 bar, 0.030 mJ/mm^2^, once/week for 3 weeks Control group: nutraceutical + splint + PT	SNCV, MNDL	VAS, BCTQs, BCTQf, Roles and Maudsley scores	Recruitment, 6 m post recruitment	NR
Ozturk et al. 2022	rESWT group: 26 LCI group: 23	Control group: 23	NR	NR	NR	NR	CTS	Median nerve	Experimental group: rESWT group: rESWT + splint 2,000 shots, 4 bar, 5 Hz, once/week for 3 weeks LCI group: LCI+ splint a local methylprednisolone (Depo-Medrol) injection of 1 mL (40 mg, without lidocaine) Control group: splint	SNAP amplitude, SNCV, CMAP amplitude, MNDL, MNCV	VAS, BCTQs, BCTQf, hand grip strength	Baseline, 12 w after treatment	The rESWT group: all patients reported pain during the procedure The LCI group: 7 patients reported pain on the day of injection
Radinmehr et al. 2019	16	16	56.0 ± 12.3	56.2 ± 8.4	9/7	10/6	Poststroke plantar flexor spasticity	Tibial nerve	Experimental group: rESWT 2,000 shots, 5 Hz, 1 bar, 0.340 mJ/mm^2^, 1 session Control group: ultrasound 1 MHz, 1.5 W/cm^2^, duration 10 min	H/M ratio, H-reflex latency	Modified MAS, AROM, PROM, PPFT, TUG	Baseline, immediately and 1 h post-treatment	No
Raissi et al. 2017	20	20	46.1 (1.95)	46.65 (2.23)	2/18	1/19	CTS	Median nerve	Experimental group: rESWT + splint 1,000 shots, 6 Hz, 1.5 bar, once/week for 3 weeks Control group: splint	SNAP amplitude, SNAP distal latency, CMAP amplitude, CMAP distal latency	VAS, The QuickDASH	Baseline, 3 w, 8 w and 12 w after the start of treatment	The rESWT group: 1 transient wrist pain after 12 w
Sağlam et al. 2022	Group 2: 42 Group 3: 41	Group 1: 42	Group 2: 53.8 ± 11.8 Group 3: 53.4 ± 10.9	Group 1: 55.8 ± 11.3	Group 2: 8/34 Group 3: 12/29	Group 1: 10/32	CTS	Median nerve	Experimental group: Group 2: rESWT + splint+ exercise 2,000 shots, 5 Hz, 4 bar, once/week for 3 weeks Group 3: PT + splint+ exercise Control group: Group 1: splint + exercise	SNCV	VAS, BCTQs, BCTQf, LANSS	Baseline, 3 w and 12 w after treatment	No
Sarhan et al. 2020	30	30	50.53 ± 3.18	52.4 ± 5.67	16/14	15/15	Diabetic neuropathic foot	Common peroneal nerve	Experimental group: ESWT + selected sensory re-education program 1,000 shots, 3 Hz, 5 bar, once/week for 3 months Control group: selected sensory re-education program	SNAP amplitude, SNAP distal latency, SNCV	The Numeric Pain Rating Scale, CSA	Before and after treatment	NR
Seok et al. 2013	15	16	54.03 ± 19.47	49.67 ± 18.83	3/12	2/14	CTS	Median nerve	Experimental group: ESWT 1,000 shots, 6 Hz, 0.09–0.29 mJ/mm^2^, 1 session Control group: LCI 1 mL of triamcinolone acetonide (40 mg)	SNAP amplitude, SNAP distal latency, SNCV CMAP amplitude, MNDL	VAS, the Levine Self-assessment Questionnaire	Baseline, 1 m and 3 m after treatment	No
Swilam et al. 2018	25	28	37.6 ± 8.5	36.8 ± 8.8	4/21	5/23	CTS	Median nerve	Experimental group: ESWT 2,500 shots, 10 Hz, 2 bar, 2 sessions with 1 week in between Control group: LCI 1 milliliter of triamcinolone acetonide (40 mg)	CAMP amplitude, MNDL, MNCV	VAS, BCTQs	Baseline, 2 w and 4 w after baseline	NR
Ulucakoy et al. 2020	Group 2: 47 Group 3: 45	Group 1: 47 Group 4: 50	Group 2: 48.4 ± 10.1 Group 3: 50 ± 8.6	Group 1: 48.1 ± 10.1 Group 4: 48.5 ± 9.8	Group 2: 8/39 Group 3: 4/41	Group 1: 7/40 Group 4: 3/47	CTS	Median nerve	Experimental group: Group 2: rESWT + splint 1,000 shots, 5 Hz, 0.05 mJ/mm^2^, once/week for 3 weeks Group 3: rESWT 1,000 shots, 5 Hz, 0.05 mJ/mm^2^, once/week for 3 weeks Control group: Group 1: splint Group 4: splint+ placebo ESWT	SNAP amplitude, SNAP distal latency, SNCV, CMAP amplitude, MNDL, MNCV	VAS, finger pinch strength, BCTQs, BCTQf, LANSS	Baseline, 3 m after treatment	No
Vongvachvasin et al. 2024	12	12	60.25 ± 6.37	58 ± 10.49	0/12	0/12	CTS	Median nerve	Experimental group: fESWT + conservative treatment + splint 1,500 shots, 0.01–0.15 mJ/mm^2^, 4–5 Hz, once/week for 3 weeks Control group: conservative treatment + splint	SNAP amplitude, SNAP distal latency, CMAP amplitude, MNDL	BCTQs, BCTQf, AUC, CSA, swelling ratio	Baseline, 3 w and 6 w after baseline	No
Wu et al. 2016	20	20	54.70 ± 7.96	57.80 ± 6.51	2/18	3/17	CTS	Median nerve	Experimental group: rESWT + splint 2,000 shots, 5 Hz, 4 bar, once/week for 3 weeks + splint Control group: Sham rESWT + splint	SNCV	VAS, BCTQs, BCTQf, CSA, finger pinch strength	Baseline, 1 w, 4 w, 8 w and 12 w after the last session	No
Xu et al. 2020	30	25	47.2 ± 1.86	46.9 ± 1.76	5/25	4/21	CTS	Median nerve	Experimental group: ESWT + splint 1,000 shots, 6 Hz, 1.5 bar, once/week for 3 weeks Control group: LCI + splint 1 milliliter (40 mg) of betamethasone	SNAP amplitude, SNAP distal latency, CMAP amplitude, CMAP distal latency	VAS, BCTQ	Baseline, 3 w, 9 w, and 12 w after the start of the treatment	No
Zhang et al. 2023	47	45	47.57 ± 3.69	46.57 ± 3.24	15/32	16/29	CTS	Median nerve	Experimental group: ESWT + nerve mobilization + splint 0.16 mJ/mm^2^, ≥2000 times, 2 times/week for 4 weeks Control group: nerve mobilization + splint	SNAP amplitude, SNCV, CMAP amplitude, MNDL	the clinical efficacy, GSS, VAS, BCTQs, BCTQf, ADL	Before and after treatment	NR

ADL, activities of daily living; AROM, active range of motion; AUC, area under curve; BCTQ, Boston Carpal Tunnel Questionnaire (s, severity and f, function); CMAP, compound muscle action potential; CSA, cross-sectional area; CTS, carpal tunnel syndrome; ESWT, extracorporeal shock wave therapy; GSS, Global Symptom Rating Scale; LANSS, Leeds Assessment of Neuropathic Symptoms and Signs; LCI, local corticosteroid injection; MAS, modified Ashworth scale; MNCV, motor nerve conduction velocity; MNDL, motor nerve distal latency; m, month; MS, multiple sclerosis; NR, not reported; PPFT, passive plantar flexor torque; PROM, passive range of motion; QuickDASH, disabilities of the arm, shoulder and hand; SNAP, sensory nerve action potential; SNCV, sensory nerve conduction velocity; SW, shock wave; TUG, the timed “up and go” test; VAS, Visual Analogue Scale; w, week.

However, two of these 24 articles were not included in the quantitative analysis due to differences in the study design. Mowafy et al. (28) and Sarhan et al. (33) both reported an ESWT duration of 3 months, which was significantly longer than the duration in other experimental groups (≤1 month), it was difficult to compare their short-term, medium-term, and long-term effects with the rest of the literature. Mowafy et al. (28) found that in patients with tarsal tunnel syndrome after burn injury, ESWT decreased the SNAP distal latency and MNDL of the medial and lateral plantar branches of the posterior tibial nerve. On the other hand, Sarhan et al. (33) observed no such significant difference in the values of conduction studies in their study.

### 3.3 Risk-of-bias assessment and certainty of evidence

**Figure 2:**
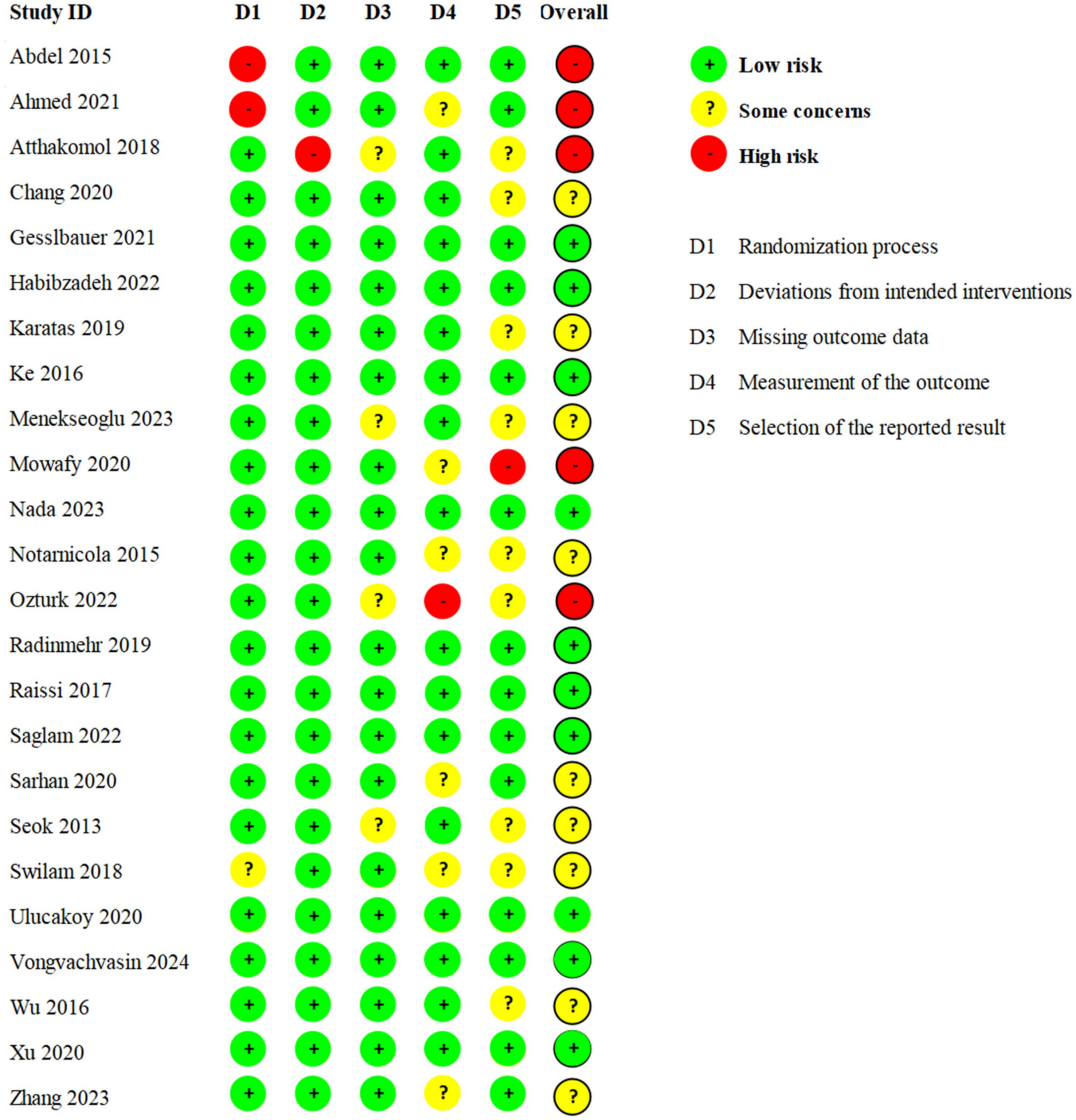
Summary of risk of bias for 24 eligible studies.

The RoB results of the 24 RCTs conducted by LY and XL were shown in Figure 2. Ten trials were at low risk, nine trials had some concerns, and five trials were at high risk. The risk factors were mainly related to the selection of the reported results. The details of the GRADE analysis for each outcome were shown in Table 2.

**Table 2:** GRADE summary of findings.

Outcome	Certainty assessment					No. of participants	MD	Certainty
	Risk of bias	Inconsistency	Indirectness	Imprecision	Other considerations			
SNAP amplitude short-term (*n* = 7)	Not serious	Very serious	Not serious	Serious	Publication bias strongly suspected	198	2.44 (−0.02, 4.90)	⊕○○○ Very low
SNAP amplitude mid-term (*n* = 7)	Serious	Serious	Not serious	Serious	Publication bias strongly suspected	216	1.19 (−0.59, 2.97)	⊕○○○ Very low
SNAP distal latency short-term (*n* = 6)	Not serious	Serious	Not serious	Serious	None	144	−0.09 (−0.21, 0.03)	⊕⊕○○ Low
SNAP distal latency mid-term (*n* = 7)	Serious	Serious	Not serious	Not serious	None	219	−0.39 (−0.52, −0.26)	⊕⊕○○ Low
SNCV short-term (*n* = 8)	Not serious	Very serious	Not serious	Not serious	Publication bias strongly suspected	289	4.36 (1.23, 7.49)	⊕○○○ Very low
SNCV mid-term (*n* = 11)	Serious	Serious	Not serious	Not serious	None	379	2.65 (1.79, 3.51)	⊕⊕○○ Low
CMAP amplitude short-term (*n* = 7)	Serious	Very serious	Not serious	Serious	Publication bias strongly suspected	176	0.84 (−0.36, 2.04)	⊕○○○ Very low
CMAP amplitude mid-term (*n* = 8)	Serious	Serious	Not serious	Serious	None	320	−0.15 (−0.65, 0.35)	⊕○○○ Very low
MNDL short-term (*n* = 9)	Not serious	Serious	Not serious	Not serious	None	248	−0.61 (−0.91, −0.30)	⊕⊕⊕○ Moderate
MNDL mid-term (*n* = 11)	Serious	Serious	Not serious	Serious	None	321	−0.36 (−0.75, 0.03)	⊕○○○ Very low
MNCV short-term (*n* = 2)	Serious	Not serious	Not serious	Serious	None	52	−0.10 (−1.89, 1.69)	⊕⊕○○ Low
MNCV mid-term (*n* = 3)	Very serious	Not serious	Not serious	Serious	None	138	0.55 (−0.36, 1.46)	⊕○○○ Very low
H/M ratio (*n* = 2)	Serious	Very serious	Not serious	Serious	Publication bias strongly suspected	31	−0.88 (−2.61, 0.86)	⊕○○○ Very low

GRADE, The Grading of Recommendations Assessment, Development and Evaluation; CMAP, compound muscle action potential; MD, mean difference; MNCV, motor nerve conduction velocity; MNDL, motor nerve distal latency; m, month; SNAP, sensory nerve action potential; SNCV, sensory nerve conduction velocity.

### 3.4 Sensitivity analyses and publication bias

The sensitivity analysis results using the leave-one-out method were presented in the Supplementary material S5. There was no publication bias in SNAP distal latency short-term and mid-term, SNCV mid-term, CMAP amplitude mid-term, MNDL short-term and mid-term, MNCV short-term and mid-term. Funnel plots showed significant publication bias for SNAP amplitude short-term and mid-term, SNCV short-term, CMAP amplitude short-term, and H/M ratio.

### 3.5 Meta-analysis results

#### 3.5.1 Post-ESWT vs. pre-ESWT

##### 3.5.1.1 SNAP amplitude

**Figure 3:**
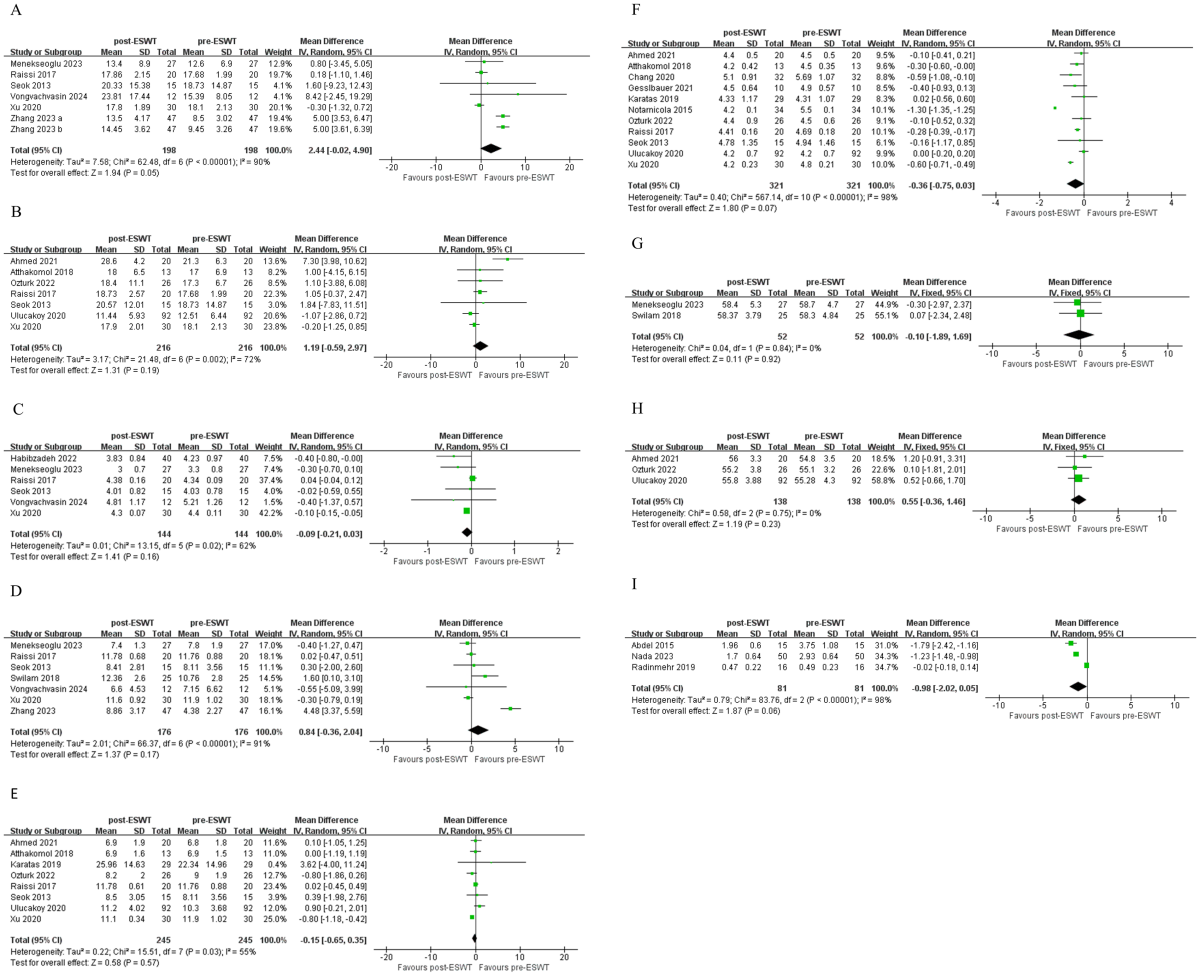
Forest plot of SNAP amplitude short-term **(A)**, SNAP amplitude mid-term **(B)**, SNAP distal latency short-term **(C)**, CMAP amplitude short-term **(D)**, CMAP amplitude mid-term **(E)**, MNDL mid-term **(F)**, MNCV short-term **(G)**, MNCV mid-term **(H)**, H/M ratio short-term **(I)**: post-ESWT compared to pre-ESWT.

There was very low certainty of evidence that changes in SNAP amplitude short-term (MD, 2.44; 95% CI: −0.02, 4.90; *I*^2^ = 90%) (Figure 3A) and mid-term (MD, 1.19; 95% CI: −0.59, 2.97; *I*^2^ = 72%) were not significantly different when comparing post-ESWT with pre-ESWT (Figure 3B).

##### 3.5.1.2 SNAP distal latency

**Figure 4:**
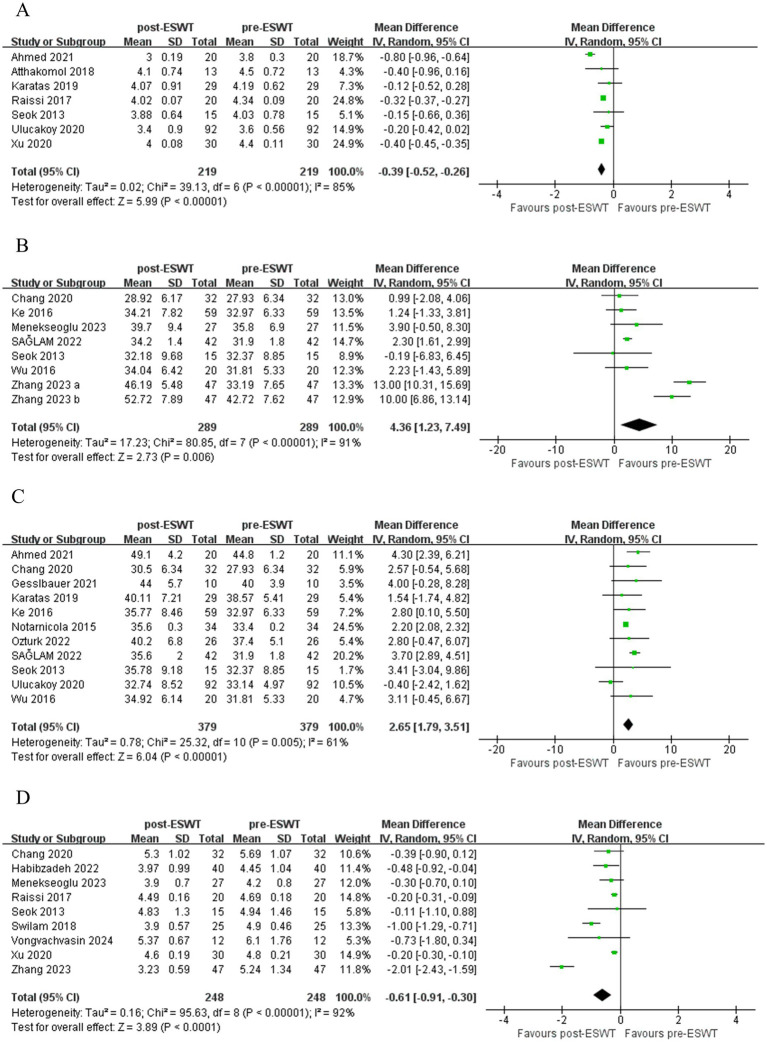
Forest plot of SNAP distal latency mid-term **(A)**, SNCV short-term **(B)**, SNCV mid-term **(C)** and MNDL short-term **(D)**: post-ESWT compared to pre-ESWT.

There was low certainty of evidence that ESWT significantly reduced SNAP distal latency mid-term compared to pre-ESWT (MD, −0.39; 95% CI: −0.52, −0.26; *I*^2^ = 85%) (Figure 4A). There was low certainty of evidence that changes in SNAP distal latency short-term were not significantly different when comparing post-ESWT with pre-ESWT (MD, −0.09; 95% CI: −0.21, 0.03; *I*^2^ = 62%) (Figure 3C).

##### 3.5.1.3 SNCV

There was very low certainty of evidence that ESWT significantly improved SNCV short-term (MD, 4.36; 95% CI: 1.23, 7.49; *I*^2^ = 91%) (Figure 4B) compared to pre-ESWT. There was low certainty of evidence that ESWT significantly improved SNCV mid-term (MD, 2.65; 95% CI: 1.79, 3.51; *I*^2^ = 61%) (Figure 4C) compared to pre-ESWT. And the changes in SNCV short-term were more obvious.

##### 3.5.1.4 CMAP amplitude

There was very low certainty of evidence that changes in CMAP amplitude short-term (MD, 0.84; 95% CI: −0.36, 2.04; *I*^2^ = 91%) (Figure 3D) and mid-term (MD, −0.15; 95% CI: −0.65, 0.35; *I*^2^ = 55%) (Figure 3E) were not significantly different when comparing post-ESWT with pre-ESWT.

##### 3.5.1.5 MNDL

There was moderate certainty of evidence that ESWT significantly reduced MNDL short-term compared to pre-ESWT (MD, −0.61; 95% CI: −0.91, −0.30; *I*^2^ = 92%) (Figure 4D). There was a very low certainty of evidence that changes in MNDL mid-term were not significantly different when comparing post-ESWT with pre-ESWT (MD, −0.36; 95% CI: −0.75, 0.03; *I*^2^ = 98%) (Figure 3F).

##### 3.5.1.6 MNCV

There was low certainty of evidence that changes in MNCV short-term (MD, −0.10; 95% CI: −1.89, 1.69; *I*^2^ = 0%) (Figure 3G) and very low certainty of evidence that changes in MNCV mid-term (MD, 0.55; 95% CI: −0.36, 1.46; *I*^2^ = 0%) were not significantly different when comparing post-ESWT with pre-ESWT (Figure 3H).

##### 3.5.1.7 H/M ratio

There was very low certainty of evidence that changes in H/M ratio short-term (MD, −0.98; 95% CI: −2.02, 0.05; *I*^2^ = 98%) were not significantly different when comparing post-ESWT with pre-ESWT (Figure 3I).

##### 3.5.1.8 H-reflex latency

Only one literature (30) included H-reflex latency, so a quantitative meta-analysis could not be performed. The results showed a significant increase in H-reflex latency compared to baseline in both the ESWT group and the ultrasound group immediately after treatment and after 1 h, but there was no statistically significant difference in the improvement between these two groups.

#### 3.5.2 ESWT vs. control

**Figure 5:**
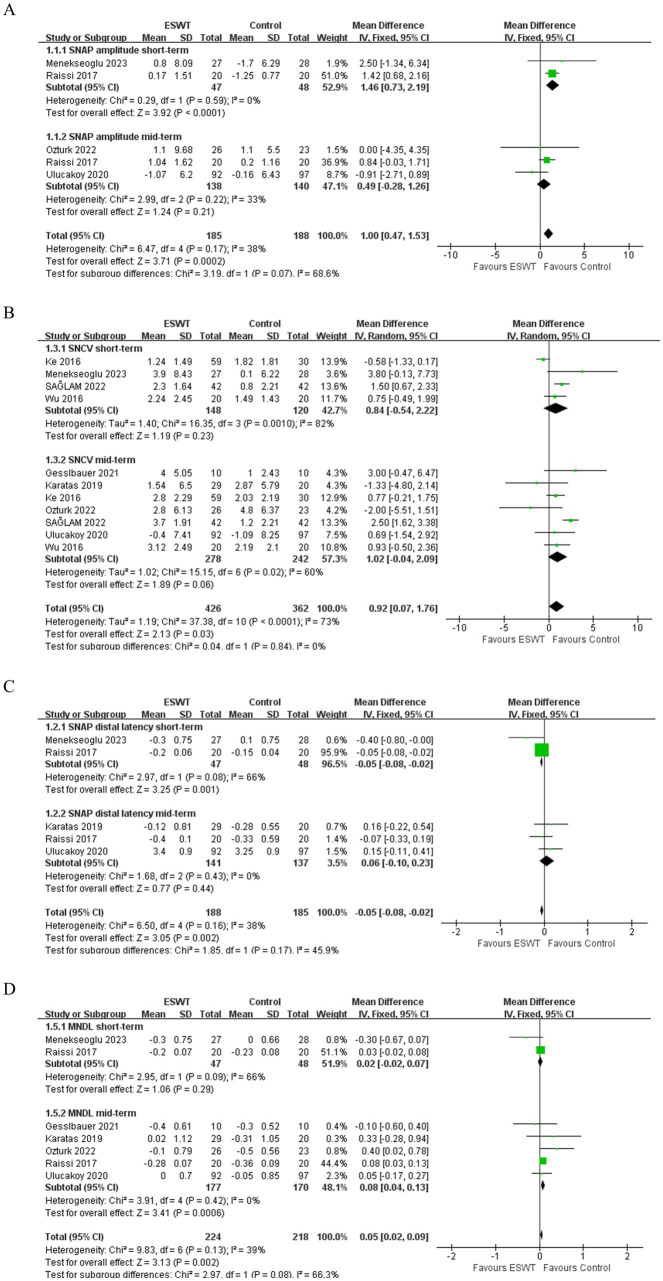
Forest plot of ESWT vs. Control: the short-term and mid-term changes in SNAP amplitude **(A)**, SNAP distal latency **(B)**, SNCV **(C)** and MNDL **(D)**.

**Figure 6:**
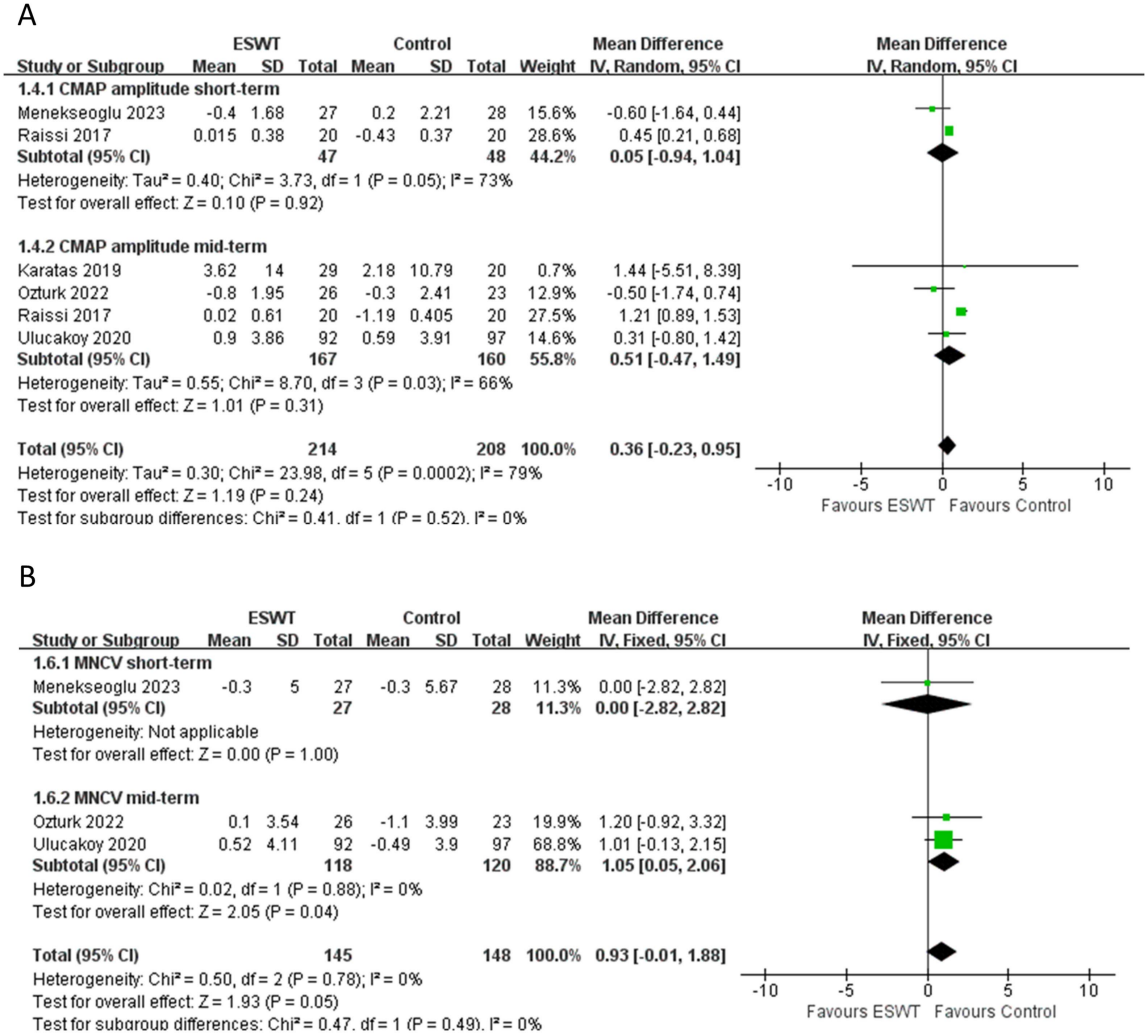
Forest plot of ESWT vs. Control: the short-term and mid-term changes in CMAP amplitude **(A)**, MNCV **(B)**.

ESWT was more effective in improving SNAP amplitude (MD, 1.00; 95% CI: 0.47, 1.53; *I*^2^ = 38%) (Figure 5A) and increasing SNCV (MD, 0.92; 95% CI: 0.07, 1.76; *I*^2^ = 73%) (Figure 5B) than Control. Evidence of low certainty suggested that ESWT was more effective in reducing SNAP distal latency than Control (MD, −0.05; 95% CI: −0.08, −0.02; *I*^2^ = 38%) (Figure 5C). However, evidence of low certainty suggested that Control was more effective in reducing MNDL than ESWT (MD, 0.05; 95% CI: 0.02, 0.09; *I*^2^ = 39%) (Figure 5D). However, there were no significant differences in the other electrophysiological parameters after the application of ESWT (Figures 6A,B).

#### 3.5.3 ESWT vs. LCI

**Figure 7:**
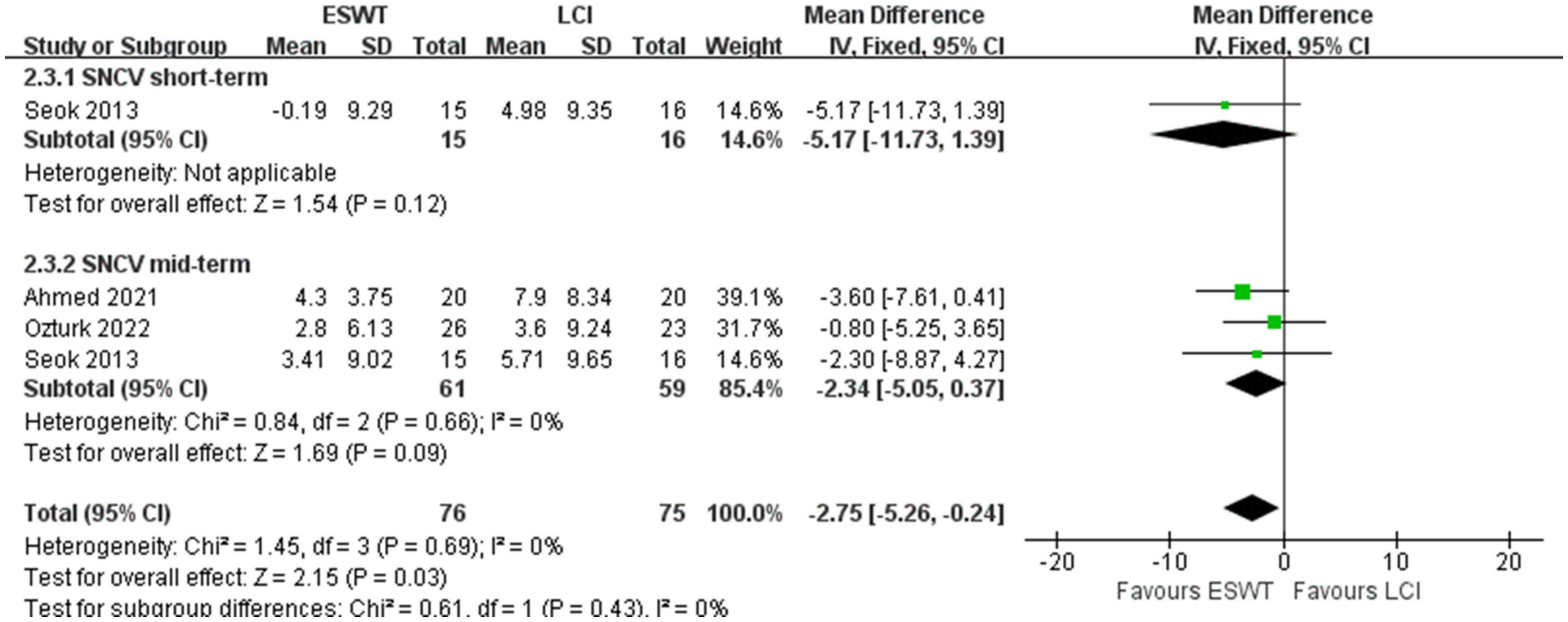
Forest plot of ESWT vs. LCI: the short-term and mid-term changes in SNCV.

**Figure 8:**
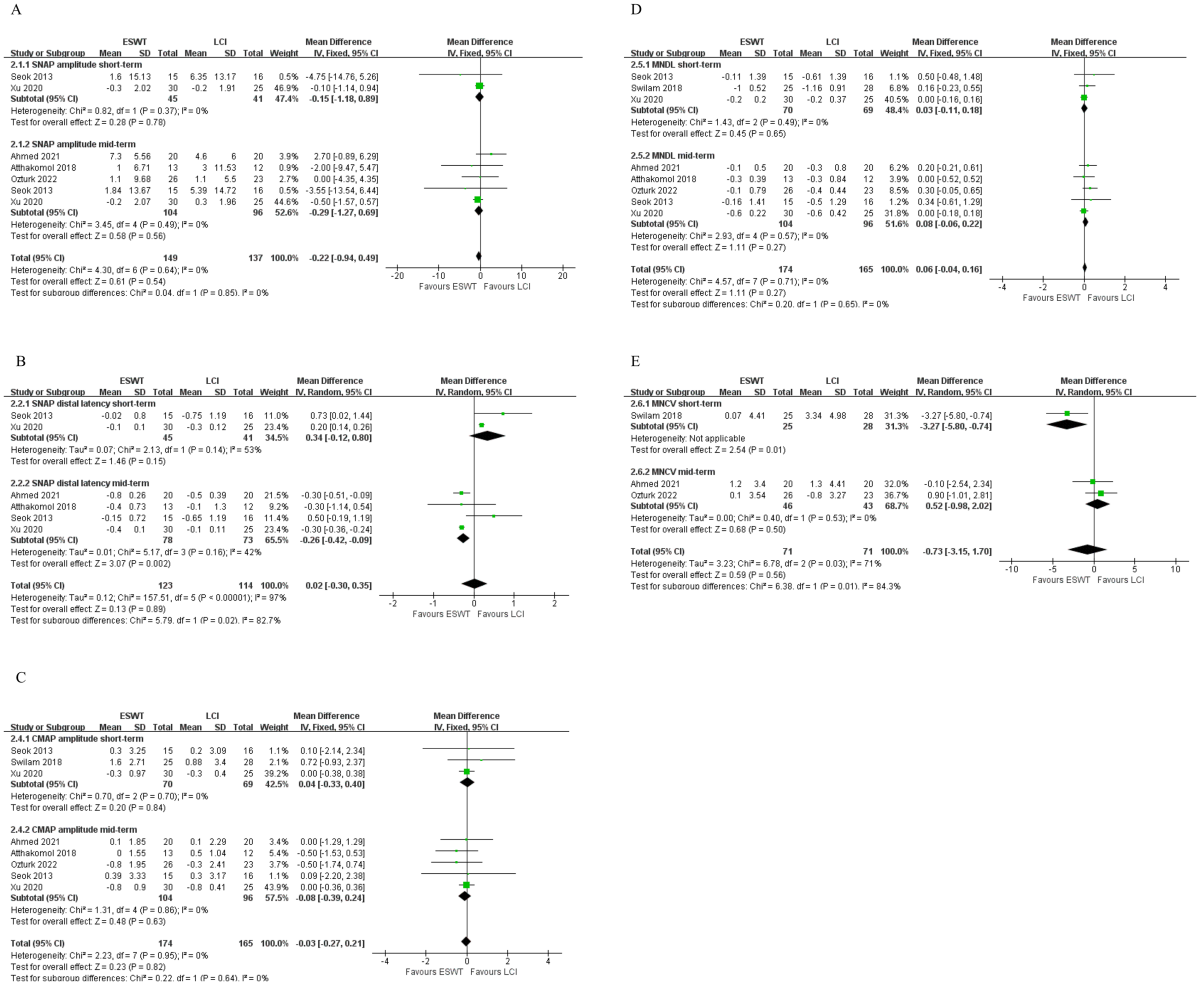
Forest plot of ESWT vs. LCI: the short-term and mid-term changes in SNAP amplitude **(A)**, SNAP distal latency **(B)**, CMAP amplitude **(C)**, MNDL **(D)**, MNCV **(E)**.

LCI was more effective than ESWT in improving SNCV (MD, −2.75; 95% CI: −5.26, −0.24; *I*^2^ = 0%) (Figure 7). However, there were no significant differences in other electrophysiological parameters after ESWT (Figures 8A–E).

#### 3.5.4 ESWT + PT vs. PT

**Figure 9:**
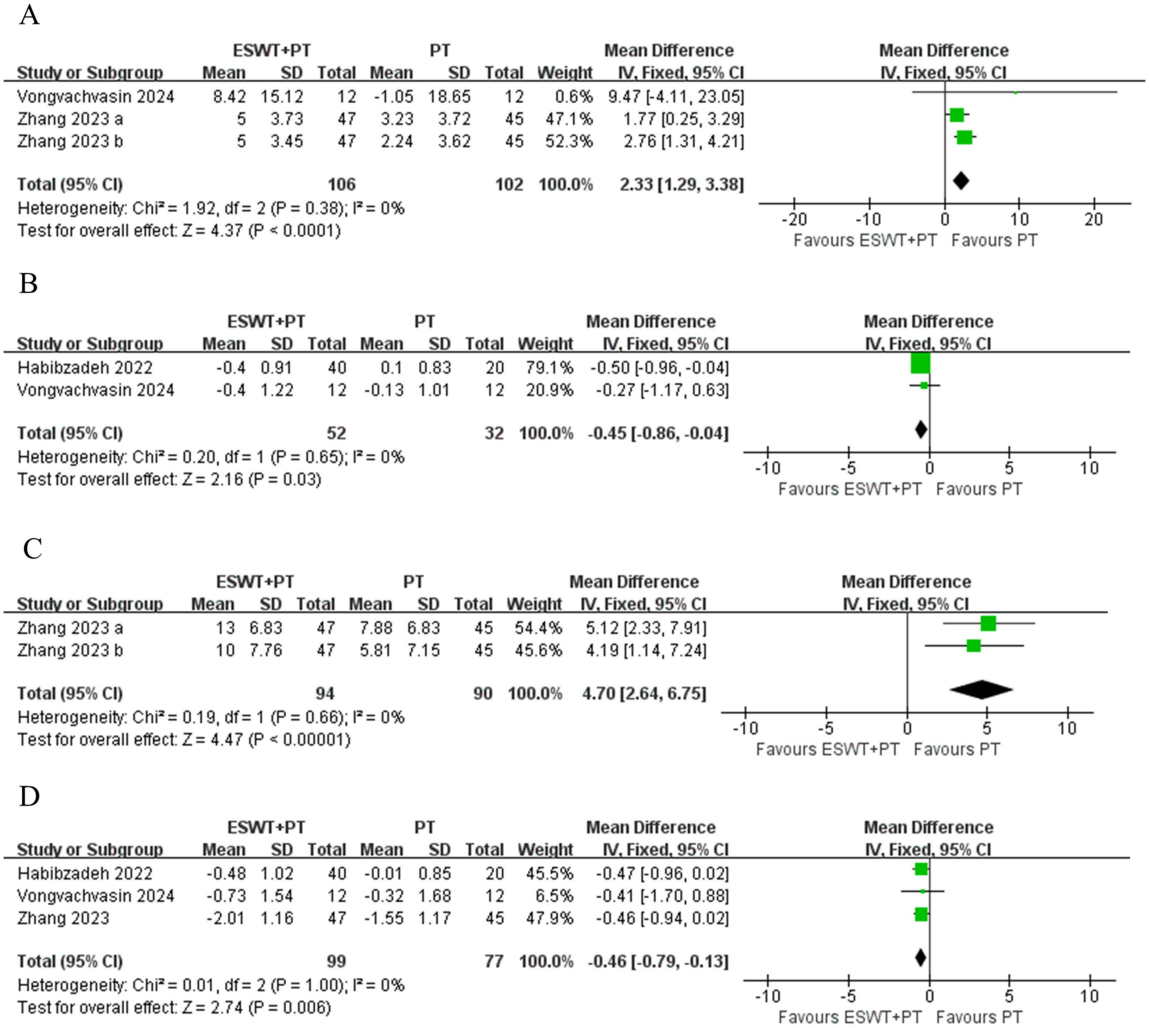
Forest plot of ESWT + PT vs. PT: the short-term changes in SNAP amplitude **(A)**, SNAP distal latency **(B)**, SNCV **(C)** and MNDL **(D)**.

**Figure 10:**
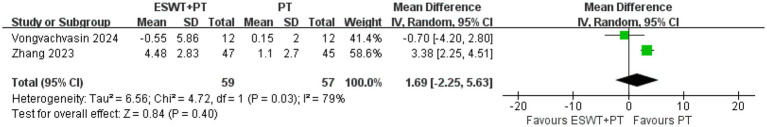
Forest plot of ESWT + PT vs. PT: the short-term changes in CMAP amplitude.

ESWT + PT was more effective in improving SNAP amplitude short-term (MD, 2.33; 95% CI: 1.29, 3.38; *I*^2^ = 0%), increasing SNCV short-term (MD, 4.70; 95% CI: 2.64, 6.75; *I*^2^ = 0%), reducing SNAP distal latency short-term (MD, −0.45; 95% CI: −0.86, −0.04; *I*^2^ = 0%), and MNDL short-term (MD, −0.46; 95% CI: −0.79, −0.13; *I*^2^ = 0%) (Figure 9) than PT. However, there were no significant differences in other electrophysiological parameters after the application of ESWT (Figure 10).

#### 3.5.5 ESWT vs. PT

Two articles compared ESWT and PT. However, these two articles used different evaluation indicators, and further quantitative analysis was not possible. Radinmehr et al. (30) compared the H/M ratio and H-reflex latency between the ESWT group and the ultrasound group in patients with poststroke plantar flexor spasticity. Compared with pre-treatment, the H-reflex latency improved, and this effect persisted for 1 h. However, the improvements were not different between groups, and there was no significant improvement in the H/M ratio. In CTS, Sağlam et al. (32) compared SNCV between PT and rESWT. There were three groups: rESWT group, PT group, and Control group. This study concluded that there was a significant increase in SNCV at 3 weeks and 3 months after treatment. In addition, rESWT showed greater improvements in all parameters than PT at 3 weeks and 3 months post-treatment.

#### 3.5.6 ESWT vs. other interventions

One RCT (29) compared ESWT and nutraceutical containing *Echinacea angustifolia*, alpha lipoic acid, conjugated linoleic acid, and quercetin (perinerv) in patients with CTS. This study showed that ESWT provided an improvement in pain, functional ability, and electrodiagnostic results until the 6-month. Both methods had a positive effect on MNDL and SNCV. However, there was no significant difference in the degree of improvement between groups. Chang et al. (17) found that combined PRP and 1-session rESWT was not superior to PRP alone for the treatment of moderate CTS at 6 months follow-up. In terms of electrodiagnostic measurements, the change in SNCV at 1 month after PRP injection was significantly lower in the intervention group than in the control group. At 3 and 6 months, this group showed greater improvements, although these improvements were not statistically significant compared to the control group. In addition, the intervention group showed a significantly greater reduction in MNDL at 3 months.

## 4 Discussion

### 4.1 Summary of main results

This systematic review showed that ESWT could reduce mid-term SNAP distal latency, improve short-term and mid-term SNCV, and reduce short-term MNDL. The results of further subgroup analyses showed that (1) ESWT appeared to be more effective than the Control group in increasing SNAP amplitude and reducing SNAP distal latency, but less effective in reducing MNDL. (2) ESWT may not be more effective than LCI in increasing SNCV. (3) ESWT + PT was more effective than PT alone in reducing SNAP amplitude and SNAP distal latency, increasing SNCV, and reducing MNDL. All the above results suggested that ESWT had an excitatory effect on nerves, which was more remarkable in the sensory nerves. Therefore, compared to other treatments, ESWT might be a useful choice for nerve lesions.

Most current ESWT studies (11, 41) evaluated the specific effects by pain score and patient-perceived global function. Although most of these studies confirmed the efficacy of ESWT in reducing pain and improving joint function, some (42, 43) showed no benefit. In the past few years, there was only one meta-analysis (12) on the neurological effects of ESWT, which was conducted with objective electrophysiological indicators. This review mainly focused on mild-to-moderate CTS patients, and low-level quality evidence indicated that the ESWT group was more effective than the control group in terms of symptom relief, functional enhancement, and electrophysiological parameters increase in both short-term (<1 m), medium-term (1–6 m) and long-term (>6 m), which significance was mainly in SNCV and MNDL. Also in this review, compared with LCI, ESWT showed better mid-term and long-term improvements in pain relief and functional recovery, and ESWT showed better mid-term (3 m) improvements only in one electrodiagnostic parameter (SNAP distal latency). However, the main purpose of this review was inconsistent with ours, which led to differences in the included trials and conclusions. In our review, there were no restrictions on the types of diseases in the subjects, and eight nerve conduction parameters were performed in the included literature (one trial on H-reflex latency was used for qualitative analysis). Furthermore, subgroup analyses were used to compare ESWT with other interventions. The ultimate goal was to evaluate the effect of ESWT on nerve conduction comprehensively.

### 4.2 Possible mechanisms

The mechanism of ESWT on nerves has not yet reached a consensus. Although it has been widely used in clinical practice for neurological disorders, especially in patients with CTS and spasticity caused by central nervous system injury (9). It may enhance the expression of a variety of growth factors (44), support the isolation and culture of Schwann cells (45), induce the synthesis of nitric oxide (NO) (46), accelerate Wallerian degeneration, and promote axonal regeneration (47). To be more specific, in recent studies on ESWT in the treatment of CP/CPPS (5), the mechanism of ESWT has been mainly related to hyperstimulation of nociceptors, tissue repair through the process of haemotransfusion, and reduction of muscle tone and stiffness. ESWT can effectively enhance the oxygenation of ulcerated tissue in treating diabetic foot ulcers (48).

ESWT can enhance the expression of growth factors such as vascular endothelial growth factor (VEGF) and Brain-Derived Neurotrophic Factor (BDNF). In recent years, several studies (44, 49, 50) had confirmed this point. Yamaya et al. (50) used low-energy ESWT to treat spinal cord injury (SCI) in 60 adult rats. The results suggested that low-energy ESWT can significantly increase the expression of VEGF and Flt-1 in the spinal cord of rats. By enhancing the neuroprotective effect of VEGF, it significantly reduced the loss of neurons in the damaged nerve tissue and minimized the secondary damage after SCI and promoted the recovery of motor function. Similarly, in the emerging field of erectile dysfunction research, low-intensity ESWT had been shown to enhance nerve regeneration and functional recovery by upregulating BDNF expression (44). Notably, this effect could remain stable at 26 days after nerve injury.

ESWT promoted the isolation and culture of Schwann cells. It is widely recognized that Schwann cells are crucial for coordinating the breakdown and resynthesis of myelin, guiding and nutritively supporting axonal regeneration (51). In a recent study by Schuh (45), an increase in the proliferation, proliferative capacity, and purity of *in vitro* Schwann cell cultures was observed with ESWT. Hercher et al. (52) also found that ESWT enhanced the regenerative capacity of Schwann cells, and in particular, the ability of sensory Schwann cells to induce neurite outgrowth and myelination was significantly enhanced.

ESWT might be associated with faster Wallerian degeneration and could accelerate the removal of degenerated axons, and improve the regenerative capacity of injured axons. Hausner et al. (47) demonstrated that low-energy ESWT could increase the rate of functional recovery at the initial stage of regeneration after sciatic nerve injury in rats. Moreover, their functional and morphological data suggested that this improved functional recovery was achieved through faster elongation of myelinated axons within the nerve after ESWT.

However, not all research on the effects of ESWT on the nervous system has been positive. Rompe et al. (53) suggested that shock wave therapy might selectively cause peripheral sensory unmyelinated nerve fiber dysfunction without affecting the nerve fibers responsible for motor function (large myelinated fibers). Kenmoku et al. (54) suggested another possible mechanism, that ESWT may selectively destroy endplates in neuromuscular junctions. A recent review by Daeschler et al. (55) argued that there was no evidence that ESWT can promote peripheral nerve regeneration. However, there were only three ESWT studies were included in Daeschler’s review, and two of them showed only temporary motor function improvement and had a high risk of bias.

Based on a previous systematic review (12), this study categorized the effects of ESWT on nerve injury into short-term (≤1 month), medium-term (1–6 months), and long-term (≥6 months) outcomes. Compared to the baseline, the results showed that: In the short-term, ESWT improved SNCV and reduced MNDL. In the mid-term, ESWT improved SNCV and reduced SNAP distal latency. Indeed, in the acute phase, progressive edema is the dominant pathology (56). The blood-nerve barrier results in endothelial capillaries that are highly permeable to fluids, proteins, and mucus substances, leading to progressive edema. The shockwaves improves nerve conduction parameters by facilitating the drainage of fluid trapped within the peripheral nerve bundles. This effect counteracts the fascicular edema primarily by reducing intraneural pressure. Instead, in the chronic phase, the shockwaves counteract the fibrotic involution of the endoneurium, release the adhesions surrounding the epineurium, and promote the vascularization and oxygenation of the nerve fascicles, improving the conduction parameters. Considering that the histopathological characteristics of peripheral nerves differ in the acute and chronic phases, this provides new insight into the biophysical effects of ESWT. Therefore, according to the main histopathological findings in different phases of neuropathies (edematous phase vs. fibrotic phase), it is necessary to adjust the treatment protocol of ESWT (e.g., frequency, energy flux, etc.).

Notably, in this review, LCI was more effective than ESWT in improving SNCV in the short and medium term (<6 m). This is consistent with the findings of Ashworth et al. (57). In the acute phase of peripheral nerve entrapment, the clinical scenario improvement of LCI is closely related to the alleviation of fascicular edema (56). However, Wang et al. (58) found that corticosteroid injection may be inferior to ESWT in long-term pain relief. In the chronic phase of the disease, when the fibrosis of the nerve and the adhesions with the surrounding soft tissues are the main histopathological findings, ESWT may be an effective treatment for patients with peripheral nerve injury (58). ESWT may result in a long-term process of pain relief and durable healing by inducing an increase in local blood flow to the diffuse chronic lesion (59) and promoting vascularisation and oxygenation of the nerve bundle (56). Nevertheless, the role of LCI at this stage is limited. Among the studies currently available for inclusion in this review, there is a lack of sufficient data to support a meta-analysis of ESWT vs. LCI in the chronic phase. We require better long-term (more than six-month) outcome studies. ESWT, like many other physical agent modalities, may have some side effects, such as pain and redness of the skin. However, this treatment had only mild and temporary side effects, with no permanent complications.

### 4.3 Clinical implications

This meta-analysis suggests that ESWT has some excitatory effect on peripheral nerves, primarily affecting sensory nerves. This finding provides evidence in support of ESWT for the treatment of peripheral neuropathy. And ESWT was better than the Control in increasing SNAP amplitude and reducing SNAP distal latency. The short-term efficacy of ESWT + PT in improving sensory and motor nerves was better than that of PT alone. Although studies have confirmed that ESWT appears to be a suitable alternative to LCI in the treatment of reducing pain and improving functional ability, it may not be more effective than LCI in terms of nerve conduction alone. Therefore, ESWT can be proposed as a viable treatment option for peripheral sensory neuropathy in clinical practice. More research is needed on the therapeutic effect of motor nerves in the future.

To date, most studies on the effects of ESWT on peripheral nerves have focused on median nerve entrapment in patients with CTS. Ashworth et al. (57) found that in the chronic phase of the disease, ESWT can be used as an effective alternative treatment modality to LCI, which is commonly used in the clinic. Similarly, in a sciatic nerve crush model, Park et al. (60) also observed that ESWT improved gait function by promoting peripheral nerve remyelination. Notably, the latest guidelines from the ISMST (6) state that spasticity, polyneuropathy, and CTS are expert indications. The mechanism of ESWT in these diseases may be related to neuromodulation, but some indications (e.g., complex regional pain syndrome, periodontal disease) are still in the experimental research stage. As a safer and non-invasive treatment, ESWT can be promoted by clinicians in their work. Although these clinical applications are promising, further applications of ESWT should be studied.

### 4.4 Future research

The applications of ESWT are promising. In future research, a deeper exploration from the following perspectives could enhance the clinical applicability of the findings. (1) Study designs: for specific diseases, the choice between rESWT and fESWT should be considered. And it is also essential to compare the impact of shockwave intensity, frequency, and intervention duration on treatment efficacy. (2) Populations: detailed studies on the severity of diseases such as CTS, which have been widely studied at present, should be carried out. However, other peripheral neuropathies (e.g., peroneal nerve entrapment), which have been less studied, deserve more attention in future research. (3) Outcomes: The observed efficacy of previous ESWT treatments has been limited to the short term, mostly less than 6 months. The long-term efficacy of treatments needs to be investigated.

### 4.5 Limitations

The studies to date have provided preliminary evidence for the effectiveness of ESWT. However, firstly, the measures are heterogeneous in this review for the following reasons: (1) the study population varied, comprising mostly elderly participants along with some children with cerebral palsy. Additionally, the diseases included differed, with a primary focus on CTS, and some involving poststroke plantar flexor spasticity, poststroke spastic equinus, etc. Sample sizes were also small across studies. All of these can lead to variability. (2) A lack of standardized research protocols led to differences in the type of ESWT, intensity and frequency of treatment, and intervention period. These posed a challenge in generalizing the findings to the whole peripheral nervous system. Secondly, nerve conduction studies are a sensitive and accurate method for assessing disease in the large myelinated nerve fibers, providing valuable diagnostic information about the underlying pathophysiology of the neuropathy. However, it does not evaluate small fibers (61). Thirdly, three studies were included in the analysis of ESWT + PT versus PT, and these trials provided only short-term results. Therefore, it remained to be seen whether the results could be translated into clinical practice as a strong recommendation. Fourthly, most of the included trials were followed up for 3 months, and only two studies were followed up for 6 months (17, 29). High-quality RCTs are still needed to determine long-term efficacy. Fifth, there is currently no standardized treatment regimen and mode for ESWT. Previous studies have found that different types of ESWT (14), different doses (62), and treatment cycles may have some influence on the effect of the treatment, and further studies on ESWT are needed. Finally, there’s not always a linear correlation between the nerve conduction parameters and the clinical findings. In this sense, in order to more accurately assess the clinical efficacy of ESWT for peripheral neuropathy, patient scores on clinical scales, such as the VAS, which reflects the level of pain, and the BCTQ-s and BCTQ-f, which assess symptom severity and functional status in CTS patients, should also be considered. This helps to provide a comprehensive reflection of the treatment effectiveness.

## 5 Conclusion

ESWT had some excitatory effect on peripheral nerves, mainly reflected in the sensory nerves. Subgroup analyses showed that ESWT was better than Control in stimulating SNAP and less effective than LCI in improving SNCV. The excitatory effect of ESWT + PT on the sensory and motor nerves was significantly better than that of PT alone.

## Data Availability

The original contributions presented in the study are included in the article/Supplementary material, further inquiries can be directed to the corresponding author.
