# Supplementary material for: Effect of extracorporeal shock wave therapy on nerve conduction: a systematic review and meta-analysis
Source: Front Neurol. 2024 Nov 22;15:1493692. doi: 10.3389/fneur.2024.1493692 (PMC11621010; doi:10.3389/fneur.2024.1493692)
Supplement: Supplementary file 1 [file Table_1.docx]

Supplementary material

1 - Search strategies for each database and registry

2 - Studies excluded during full-text examination with reasons

3 - Description of outcomes and outcome measures

4 - Characteristics of other included studies (ongoing)

5 - Sensitivity analyses

**Supplementary material 1 -** Search strategies for each database and registry

TABLE 1. PubMed August 20, 2024.

| Concept | Query | Hits |
| --- | --- | --- |
| #1 | (Extracorporeal Shockwave[Title/Abstract]) OR (Extracorporeal Shock Wave[Title/Abstract]) | 7725 |
| #2 | (ESWT[Title/Abstract]) OR (ESW[Title/Abstract]) | 1781 |
| #3 | (#1) OR (#2) | 8133 |
| #4 | (nerve[Title/Abstract]) OR (nerves[Title/Abstract]) | 490515 |
| #5 | (#3) AND (#4) | 201 |

TABLE 2. Web of Science August 20, 2024.

| Concept | Query | Hits |
| --- | --- | --- |
| #1 | TS=("extracorporeal shockwave*" OR "extracorporeal shock wave*" OR "ESWT" OR "ESW") | 8901 |
| #2 | TS=("nerve" OR "nerves") | 529534 |
| #3 | #10 AND #4 | 268 |

TABLE 3. Cochrane Library August 20, 2024.

| Concept | Query | Hits |
| --- | --- | --- |
| #1 | (Extracorporeal Shockwave or Extracorporeal Shock Wave or ESWT or ESW):ti,ab,kw | 2897 |
| #2 | (nerve or nerves):ti,ab,kw | 49732 |
| #3 | #1 and #2 | 153 |

TABLE 4. Embase August 20, 2024.

| Concept | Query | Hits |
| --- | --- | --- |
| #1 | 'extracorporeal shockwave' OR 'extracorporeal shock wave'/exp OR 'extracorporeal shock wave' OR 'eswt' OR 'esw' | 17043 |
| #2 | 'nerve' | 1565570 |
| #3 | #1 AND #2 | 507 |
| #4 | #3 AND 'article'/it | 253 |

**Supplementary material 2 - Studies excluded during full-text examination with reasons**

| **No.** | **Study (first author, year)** | **Reason for exclusion** |
| --- | --- | --- |
| 1 | Bolt 2004 | Animal experiment |
| 2 | Daliri 2015 | Not randomized controlled study |
| 3 | Guidetti 2024 | Not randomized controlled study |
| 4 | Hausner 2012 | Animal experiment |
| 5 | Heinzel 2022 | Animal experiment |
| 6 | Karatas 2017 | Same experiment |
| 7 | Kenmoku 2012 | Animal experiment |
| 8 | Kenmoku 2018 | Animal experiment |
| 9 | Kim 2022 | Animal experiment |
| 10 | Lee 2023 | Animal experiment |
| 11 | Luh 2018 | Animal experiment |
| 12 | Manganotti 2005 | Not randomized controlled study |
| 13 | Manganotti 2012 | Not randomized controlled study |
| 14 | Marinelli 2015 | Evaluation indicators missing control group comparisons |
| 15 | Notarnicola 2018 | Not randomized controlled study |
| 16 | Park 2019 | Animal experiment |
| 17 | Rashad 2020 | Incomplete data reporting |
| 18 | Seo 2021 | Animal experiment |
| 19 | Sohn 2011 | Not randomized controlled study |
| 20 | Wu 2007 | Animal experiment |
| 21 | Wu 2008 | Animal experiment |
| 22 | Vongvachvasin 2023 | Same experiment |
| 23 | Zong 2023 | Not randomized controlled study |

**Supplementary material 3 - Description of outcomes and outcome measures**

| Outcome | ESWT vs baseline | ESWT VS Control | ESWT VS LCI | ESWT+PT VS PT | ESWT VS PT | ESWT vs. Other Interventions |
| --- | --- | --- | --- | --- | --- | --- |
| SNAP amplitude | Ahmed 2021 | Menekseoglu 2023 | Ahmed 2021 | Vongvachvasin 2024 |  |  |
|  | Atthakomol 2018 | Ozturk 2022 | Atthakomol 2018 | Zhang 2023 |  |  |
|  | Menekseoglu 2023 | Raissi 2017 | Ozturk 2022 |  |  |  |
|  | Ozturk 2022 | Ulucakoy 2020 | Seok 2013 |  |  |  |
|  | Raissi 2017 |  | Xu 2020 |  |  |  |
|  | Seok 2013 |  |  |  |  |  |
|  | Ulucakoy 2020 |  |  |  |  |  |
|  | Vongvachvasin 2024 |  |  |  |  |  |
|  | Xu 2020 |  |  |  |  |  |
|  | Zhang 2023 |  |  |  |  |  |
| SNAP distal latency | Ahmed 2021 | Karatas 2019 | Ahmed 2021 | Habibzadeh 2022 |  |  |
|  | Atthakomol 2018 | Menekseoglu 2023 | Atthakomol 2018 | Vongvachvasin 2024 |  |  |
|  | Habibzadeh 2022 | Raissi 2017 | Seok 2013 |  |  |  |
|  | Karatas 2019 | Ulucakoy 2020 | Xu 2020 |  |  |  |
|  | Menekseoglu 2023 |  |  |  |  |  |
|  | Raissi 2017 |  |  |  |  |  |
|  | Seok 2013 |  |  |  |  |  |
|  | Ulucakoy 2020 |  |  |  |  |  |
|  | Vongvachvasin 2024 |  |  |  |  |  |
|  | Xu 2020 |  |  |  |  |  |
| SNCV | Ahmed 2021 | Gesslbauer 2021 | Ahmed 2021 | Zhang 2023 | Sağlam 2022 | Chang 2020 |
|  | Chang 2020 | Karatas 2019 | Ozturk 2022 |  |  | Notarnicola 2015 |
|  | Gesslbauer 2021 | Ke 2016 | Seok 2013 |  |  |  |
|  | Karatas 2019 | Menekseoglu 2023 |  |  |  |  |
|  | Ke 2016 | Ozturk 2022 |  |  |  |  |
|  | Menekseoglu 2023 | Sağlam 2022 |  |  |  |  |
|  | Notarnicola 2015 | Ulucakoy 2020 |  |  |  |  |
|  | Ozturk 2022 | Wu 2016 |  |  |  |  |
|  | Sağlam 2022 |  |  |  |  |  |
|  | Seok 2013 |  |  |  |  |  |
|  | Ulucakoy 2020 |  |  |  |  |  |
|  | Wu 2016 |  |  |  |  |  |
|  | Zhang 2023 |  |  |  |  |  |
| CMAP amplitude | Ahmed 2021 | Karatas 2019 | Ahmed 2021 | Vongvachvasin 2024 |  |  |
|  | Atthakomol 2018 | Menekseoglu 2023 | Atthakomol 2018 | Zhang 2023 |  |  |
|  | Karatas 2019 | Ozturk 2022 | Ozturk 2022 |  |  |  |
|  | Menekseoglu 2023 | Raissi 2017 | Seok 2013 |  |  |  |
|  | Ozturk 2022 | Ulucakoy 2020 | Swilam 2018 |  |  |  |
|  | Raissi 2017 |  | Xu 2020 |  |  |  |
|  | Seok 2013 |  |  |  |  |  |
|  | Swilam 2018 |  |  |  |  |  |
|  | Ulucakoy 2020 |  |  |  |  |  |
|  | Vongvachvasin 2024 |  |  |  |  |  |
|  | Xu 2020 |  |  |  |  |  |
|  | Zhang 2023 |  |  |  |  |  |
| MNDL | Ahmed 2021 | Gesslbauer 2021 | Ahmed 2021 | Habibzadeh 2022 |  | Chang 2020 |
|  | Atthakomol 2018 | Karatas 2019 | Atthakomol 2018 | Vongvachvasin 2024 |  | Notarnicola 2015 |
|  | Chang 2020 | Menekseoglu 2023 | Ozturk 2022 | Zhang 2023 |  |  |
|  | Gesslbauer 2021 | Ozturk 2022 | Seok 2013 |  |  |  |
|  | Habibzadeh 2022 | Raissi 2017 | Swilam 2018 |  |  |  |
|  | Karatas 2019 | Ulucakoy 2020 | Xu 2020 |  |  |  |
|  | Menekseoglu 2023 |  |  |  |  |  |
|  | Notarnicola 2015 |  |  |  |  |  |
|  | Ozturk 2022 |  |  |  |  |  |
|  | Raissi 2017 |  |  |  |  |  |
|  | Seok 2013 |  |  |  |  |  |
|  | Swilam 2018 |  |  |  |  |  |
|  | Ulucakoy 2020 |  |  |  |  |  |
|  | Vongvachvasin 2024 |  |  |  |  |  |
|  | Xu 2020 |  |  |  |  |  |
|  | Zhang 2023 |  |  |  |  |  |
| MNCV | Ahmed 2021 | Menekseoglu 2023 | Ahmed 2021 |  |  |  |
|  | Menekseoglu 2023 | Ozturk 2022 | Ozturk 2022 |  |  |  |
|  | Ozturk 2022 | Ulucakoy 2020 | Swilam 2018 |  |  |  |
|  | Swilam 2018 |  |  |  |  |  |
|  | Ulucakoy 2020 |  |  |  |  |  |
| H/M | Abdel 2015 | Nada 2023 |  | Abdel 2015 | Radinmehr 2019 |  |
|  | Radinmehr 2019 |  |  |  |  |  |
|  | Nada 2023 |  |  |  |  |  |
| H-reflex | Radinmehr 2019 |  |  |  | Radinmehr 2019 |  |

Abbreviations: CMAP, compound muscle action potential; ESWT, Extracorporeal shock wave therapy; LCI, Local corticosteroid injection; MNCV, motor nerve conduction velocity; MNDL, motor nerve distal latency; PT, physical therapy; SNAP, sensory nerve action potential; SNCV, sensory nerve conduction velocity.

**Supplementary material 4 - Characteristics of other included studies (ongoing)**

| **Study title** | **Trial ID** | **Study Start Date** | **Target Sample Size** | **Intervention** | **Control** | **Outcome** | | **Follow-up** |
| --- | --- | --- | --- | --- | --- | --- | --- | --- |
|  |  |  |  |  |  | **Electrodiagnostic studies** | **Other** |  |
| Effectiveness of Shock Wave Therapy for Upper Limb Spasticity | NCT04316026 | 2019-09-02 | 48 | ESWT | sham ESWT | H reflex | MAS, Tardieu scale, elastic and viscous stiffness of the wrist, muscle stiffness, joint range of motion, strength assessment, muscle stiffness at the wrist, Box and block test, Fugl Meyer Assessment, Abilhand scale, Wolff Motor Function Test, VAS | 2 weeks |
| Multiphasic Neuroplasticity Based Training Protocol With Shock Wave Therapy For Post Stroke Spasticity | NCT05405140 | 2022-05-20 | 32 | A: SWT + conventional rehabilitation training B: MNTP + conventional rehabilitation training C: SWT+ MNTP + conventional rehabilitation training | D: conventional rehabilitation training | F wave , H reflex | MAS, Resting joint angle, Muscle length, Muscle thickness, Muscle pennation angle, Time up and Go, Burg Balance scale, Functional independence measure, Barthal index, Rivermead Mobility Index | 6 weeks |
| Efficacy of EPSW Plus HILT on Carpal Tunnel Syndrome Post Burn Injury | NCT05876442 | 2023-05-20 | 120 | Group A: ESWT + HILT + PT Group B: ESWT + PT Group C: HILT + PT | PT | Median Nerve Conduction Study | VAS, BCTQ, muscle strength assessment | 2 months |
| Mechanism of Action of Focal Extracorporeal Shock Waves as a Treatment of Upper Limb Stroke Spasticity: a Pilot Study | NCT06311526 | 2023-05-30 | 12 | ESWT | None | H/M ratio, F waves | T reflex, upper limb dexterity measures, ultrasound arm and forearm assessment | 84 days |
| Effect of Shockwave Therapy on Post-laminectomy Fibrosis Low Back Pain Patients | NCT05887024 | 2023-06-01 | 60 | rESWT+PT | PT | Sensory Nerve Conduction Study | VAS, pain algometry, lumbar range of motion, the Oswestry Disability Index | 4 weeks |
| Myo-Electrical and Nerve Root Function Response to Focused Extracorpeal Shock Wave in Cervical Radiculopathy | NCT06102304 | 2023-09-06 | 38 | fESWT+ PT the taut band: 700 shots, 0.056 mJ/mm^2^, 10 Hz surrounding the taut band: 300 shots, 0.056 mJ/mm^2^, 10 Hz 2 sessions/week for 4 weeks | sham ESWT+PT | SNAP amplitude, SNAP distal latency | pain pressure threshold, hand grip strength, numeric rating scale | 4 weeks |
| Efficacy of Extracorporeal Shock Wave Therapy on Carpal Tunnel Syndrome Post Mastectomy Lymphedema | NCT06131515 | 2023-11 | 68 | ESWT+PT The treated area was parallel to the median nerve from the pisiform level to 2cm proximal to the inlet of the carpal tunnel with equal diffusion of 800, 900, 1000, 1100 shots from first to fourth session respectively 4 bar, 5 Hz, 2 sessions/week for 4 weeks | sham ESWT | MNDL, SNAP distal latency | limb volume, VAS, BCTQ | 4 weeks |
| Adding Shock Wave Therapy to Kinesio Taping Improves Carpal Tunnel Syndrome in Physical Therapist Females | NCT06214286 | 2024-03-20 | 100 | shock wave therapy + PT | PT | SNAP distal latency, MNDL | VAS, BCTQ, handgrip strength | 12 weeks |

Abbservations: BCTQ, Boston Carpal Tunnel Questionnaire; CMAP, compound muscle action potential; CSA, cross-sectional area; EPSW, Extracorporeal Shock Wave; ESWT, Extracorporeal shock wave therapy; fESWT, focused ESWT; rESWT, radial ESWT; HILT, High-intensity Laser Therapy; MAS, Modified Ashworth Scale; MNCV, motor nerve conduction velocity; MNDL, motor nerve distal latency; MNTP, multiphasic neuroplasticity based training protocol; PT, physical therapy; SNAP, sensory nerve action potential; SNCV, sensory nerve conduction velocity; VAS, Visual Analogue Scale.

**Supplementary material 5 - Sensitivity analyses**

| Outcome | Leave-one-out | Sensitivity Analysis | |
| --- | --- | --- | --- |
| SNAP amplitude short-term | Xu 2020 | (MD,3.13; 95% CI:0.47,5.79; I^2^=86%) | The heterogeneity was reduced, and ESWT significantly improved SNAP amplitude short-term compared to pre-ESWT. |
| SNAP amplitude mid-term | Ahmed 2021 | (MD,0.06; 95% CI:-0.69,0.80; I^2^=0%) | The heterogeneity was disappeared and the pooled effect size was lowered. While there was still no statistically significant difference between groups. |
| SNAP distal latency short-term | Raissi 2017 | (MD,-0.11; 95% CI:-0.15,-0.06; I^2^=0%) | The heterogeneity was disappeared, and ESWT significantly reduced SNAP distal latency short-term compared to pre-ESWT. |
|  | Xu 2020 | (MD,-0.14; 95% CI:-0.37,0.09; I^2^=47%) | The heterogeneity was reduced. |
| SNAP distal latency mid-term | Ahmed 2021 | (MD,-0.34; 95% CI:-0.41,-0.27; I^2^=45%) | The heterogeneity was reduced. |
| SNCV short-term | Zhang 2023 a | (MD,3.07; 95% CI:0.91,5.23; I^2^=76%) | The heterogeneity was reduced and the pooled effect size was lowered. |
|  | Zhang 2023 b | (MD,3.54; 95% CI:0.38,6.70; I^2^=90%) | The pooled effect size was lowered. |
| SNCV mid-term | Notarnicola 2015 | (MD,2.76; 95% CI:1.68,3.85; I^2^=45%) | The heterogeneity was reduced and the pooled effect size was improved. |
|  | SAĞLAM 2022 | (MD,2.35; 95% CI:1.52,3.17; I^2^=29%) | The heterogeneity was reduced and the pooled effect size was lowered. |
|  | Ulucakoy 2020 | (MD,2.99; 95% CI:2.17,3.80; I^2^=52%) | The heterogeneity was reduced and the pooled effect size was improved. |
| CMAP amplitude short-term | Zhang 2023 | (MD,-0.06; 95% CI:-0.46,0.35; I^2^=22%) | The heterogeneity was reduced and the pooled effect size was lowered. |
| CMAP amplitude mid-term | Ulucakoy 2020 | (MD,-0.32; 95% CI:-0.77,0.14; I^2^=42%) | The heterogeneity was reduced and the pooled effect size was lowered. |
|  | Xu 2020 | (MD,0.04; 95% CI:-0.32,0.40; I^2^=0%) | The heterogeneity was disappeared and the pooled effect size was improved. |
| MNDL short-term | Zhang 2023 | (MD,-0.40; 95% CI:-0.60,-0.20; I^2^=77%) | The heterogeneity was reduced and the pooled effect size was improved. |
| MNDL mid-term | Notarnicola 2015 | (MD,-0.27; 95% CI:-0.44,-0.09; I^2^=77%) | The heterogeneity was reduced and the pooled effect size was improved. |
